# First Records and Expanding Distribution of a Small Big-Headed Ant, *Pheidole parva*, in Florida, USA

**DOI:** 10.1007/s13744-026-01416-4

**Published:** 2026-07-21

**Authors:** Marina S. Ascunce, Douglas B. Booher, Aaron C. Stoll, Airlan San Juan, Agnes Lee, Sanford D. Porter

**Affiliations:** 1 Center for Medical, Agricultural, and Veterinary Entomology (CMAVE), Imported Fire Ant and Household Insects (IFAHI) Unit, USDA-Agricultural Research Service, Gainesville, FL USA; 2 Univ of Georgia, Athens, GA USA; 3 Pensacola, FL USA; 4 Keyapa.com, New Brunswick, NJ USA

**Keywords:** Invasion, Ants, *Pheidole*, Florida, Non-native, Genetic diversity

## Abstract

*Pheidole parva* Mayr (1865) is a small ubiquitous ant native to the Indomalayan region where it is considered a pest in healthcare facilities. This tramp ant was first reported outside its native range more than 100 years ago in the Seychelles Islands. Since then, it has been found in other areas of the Old World, where it was likely accidentally introduced via trading ships. In the 2000s, this ant was detected in Japan (2001), the Arabian Peninsula (2009), and recently in the Mediterranean island of Cyprus (2023) and Lebanon (2025). Here, we describe its first occurrence in North America, in the state of Florida where both Nearctic and Neotropical realms meet. Our findings are based on recent *Pheidole parva* specimens collected throughout the state, curated iNaturalist observations, Antweb data, and DNA barcoding based on the mitochondrial cytochrome oxidase subunit I (COI) gene. Among the 26 *P. parva* specimens analyzed from Florida, we found three different COI haplotypes. Two of them were also found in other introduced areas; however, one of the haplotypes has only been detected in a native population. The three haplotypes were distributed throughout the state with no clear geographical structure, consistent with multiple introductions and broad establishment of this non-native ant in Florida.

**Supplementary Information:**

The online version contains supplementary material available at 10.1007/s13744-026-01416-4.

## Introduction

Representing the southeastern tip of North America, the state of Florida is mostly situated on a peninsula. The latitudinal temperature gradient ranges from tropical in southern Florida, to subtropical and temperate in the northern limits. There are more than 80 terrestrial ecosystems from wetlands, coastal dunes, swamps, and pine flatwoods among others that house a large biodiversity with a high proportion of endemic taxa (Stein et al. 2000). However, anthropogenic disturbances through urban development, agriculture, mining, drainage projects, artificial channels, and expansion of transportation networks among others have led to loss of natural habitat and fragmentation, which has resulted in the loss of natural biodiversity (Volk et al. 2017; Ahmed and Weeks 2025; Skelley et al. 2025). Furthermore, what makes Florida so unique and biodiversity rich, it also makes it susceptible to invasive species, placing Florida as a global hotspot of invasive species for multiple taxonomic groups such as plants, birds, reptiles, and fishes, including ants (Dawson et al. 2017; Skelley et al. 2025). Important drivers of non-native species introductions are the movements of goods as well as humans (Hulme 2009). This is particularly important regarding plants as 85% of all imported plant materials such as fruits, cut flowers, and ornamental plants enter the USA through Miami (Frank and McCoy 1995; Caton et al. 2006), and plant materials are known for their role in transporting invasive arthropod pests (Liebhold et al. 2012), particularly ants (Wong et al. 2023). This high volume of global trade plus anthropogenic disturbances may facilitate ant invasions, and may be related to Florida currently hosting the largest non-native ant fauna in the continental USA, where 63 of 229 species are non-native (Deyrup et al. 2000; Booher et al. 2023). This non-native ant fauna is also among the largest ones in the world (King and Porter 2007; Wong et al. 2023) underscoring the importance of early detection of non-native ants in the state, especially those with known invasive potential. Thus, the recent detection of the small big-headed ant, *Pheidole parva* Mayr (1865) , a species not previously reported from the state, occurring in northern, central, and southern Florida prompted coordinated efforts among the authors to confirm its identity and assess its establishment in the region. In this study, we report its occurrence for the first time in North America with specimens collected by the authors in the state of Florida. To assess the extent of *P. parva* in North America, we combined our statewide sampling efforts in Florida with records from AntWeb and the community-science platform iNaturalist. This survey revealed observations in Louisiana and in the Bahamas; however, in the absence of collected specimens, these remain provisional and require voucher-based confirmation before being treated as first records in those regions.

**Fig. 1:**
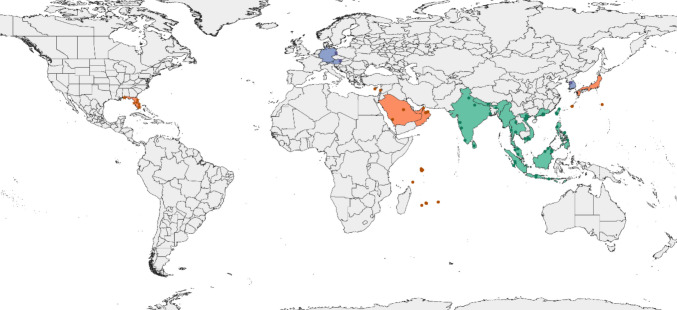
Worldwide distribution of *Pheidole parva*. Green shading indicates native range in the Indomalayan range. Orange shading shows regions where the species has been collected outdoors, while blue shading denotes indoor detections. Only voucher-confirmed records from this study, published articles, and AntWeb are included in the map. iNaturalist observations, including indoor detections, were not mapped

*Pheidole parva* Mayr (1865) belongs to the *Pheidole rinae* Emery complex, which comprises five morphologically similar taxa native to the Indomalayan region (Eguchi et al. 2007). Members of this group exhibit subtle morphological differences, making species delimitation challenging. Both Eguchi et al. (2007) and Fisher and Fisher (2013) concluded with the following synonyms for *P. parva*: *Pheidole parva* var. *decanica* Forel, 1902; *Pheidole flavens* var. *farquharensis* Forel 1907; *Pheidole sauteri* Wheeler, 1909; *Pheidole rinae* var. *mala* Forel, 1911; *Pheidole rinae* r. *tipuna* Forel, 1912; *Pheidole bugi* Wheeler, 1919; *Pheidole tardus* Donisthorpe, 1947. The presumptive native distribution of *Pheidole parva* includes Borneo, India, Indonesia, Malaysia, Myanmar, Nepal, Philippines, Singapore, Sri Lanka (type locality), Taiwan, Thailand, Vietnam, Hong Kong, and southern coastal provinces of China (Eguchi et al. 2007; AntWeb n.d.) (Fig. 1). In Singapore, where *P. parva* is native, the species can reach pest status and has been reported as a nuisance in healthcare facilities (Man and Lee 2014). Outside its native range, more than 100 years ago, Forel (1907) reported this ant in the Seychelles Islands in the Southwest Indian Ocean (SWIO), where it was probably introduced via trading ships. Subsequent surveys of multiple islands in the region by Fisher and Fisher (2013) found *P. parva* to be widespread and ubiquitous. The species occurred across a broad variety of habitats including gardens, mangroves, coastal scrub, mixed forests, rainforests, and disturbed environments, highlighting its potential to invade new areas through human commerce. Over the past two decades, *P. parva* has been detected in a growing number of geographic regions, including East Asia (Japan, 2001), the Arabian Peninsula (2009), and more recently, the Mediterranean region, with records from the island of Cyprus (2023) and in Lebanon (2025) (Fig. 1). In addition, this ant was found in indoor settings in Europe (Germany and Austria, 2008), and more recently in South Korea (2025). All corresponding references are summarized in Table 1.

**Table 1:** *Pheidole parva* first detection records in introduced areas

Geographic region	Geographic area/country	Island/state	Detection year	References
Southwest	Seychelles	Farquhar	1907	Forel (1907)
Indian Ocean	Islands	North Island	2000	Fischer and Fisher (2013)
(SWIO)		Aride Island	2005	Fischer and Fisher (2013)
		Cousine Island	2008	Fischer and Fisher (2013)
		Bird Island	2010	Fischer and Fisher (2013)
		Conception Island	2010	Fischer and Fisher (2013)
		Curieuse Island	2010	Fischer and Fisher (2013)
		Félicité Island	2010	Fischer and Fisher (2013)
		La Digue Island	2010	Fischer and Fisher (2013)
		Mahé Island	2010	Fischer and Fisher (2013)
		Praslin Island	2010	Fischer and Fisher (2013)
		Silhouette Island	2010	Fischer and Fisher (2013)
	Mascarene	Mauritius Island	1946	Donisthorpe (1946)
	Islands	Ile aux Aigrettes	2005	Fischer and Fisher (2013)
		Rodrigues Island	2005	Fischer and Fisher (2013)
		La Réunion	2024	Carval et al. (2026)
East Asia	Japan	Ryukyus Islands	2001	Eguchi et al. (2007)
		Ogasawara Islands	2003	Eguchi et al. (2007)
		Honshu Island	2021	Terayma et al. (2021)
	South Korea^**1**^	Seoul	2020	Dong et al. (2025)
Arabian	United Arab	Wadi Wurayah	2009	Fischer and Fisher (2013)
Peninsula	Emirates (UAE)			
	Kingdom of Saudi	Al Bahah	2011	Fischer and Fisher (2013)
	Arabia (KSA)			
	Oman	Nakhl	2016	Sharaf et al. (2018)
	Kingdom of Bahrain	Budaiya	2022	Sharaf et al. (2024)
Mediterranean	Cyprus Island	Asomatos	2023	Demetriou et al. (2025)
Sea				
	Lebanon	Keserwan-Jbeil	2025	Schifani and Massaad (2026)
Europe	Austria^**1**^			Eguchi (2008)
	Germany^**1**^			Eguchi (2008)
North	USA	Florida	2023	This report
America		Maryland^**2**^	2025	iNaturalist

^1^Indoor record verified by voucher specimens (included in map)

^2^Indoor record from Maryland (2025) based solely on an iNaturalist observation (“In an enclosed butterfly exhibit, with butterflies sourced from breeders in Florida and Colorado”); not included in the map due to lack of voucher confirmation

## Material and methods

### Sampling

**Fig. 2:**
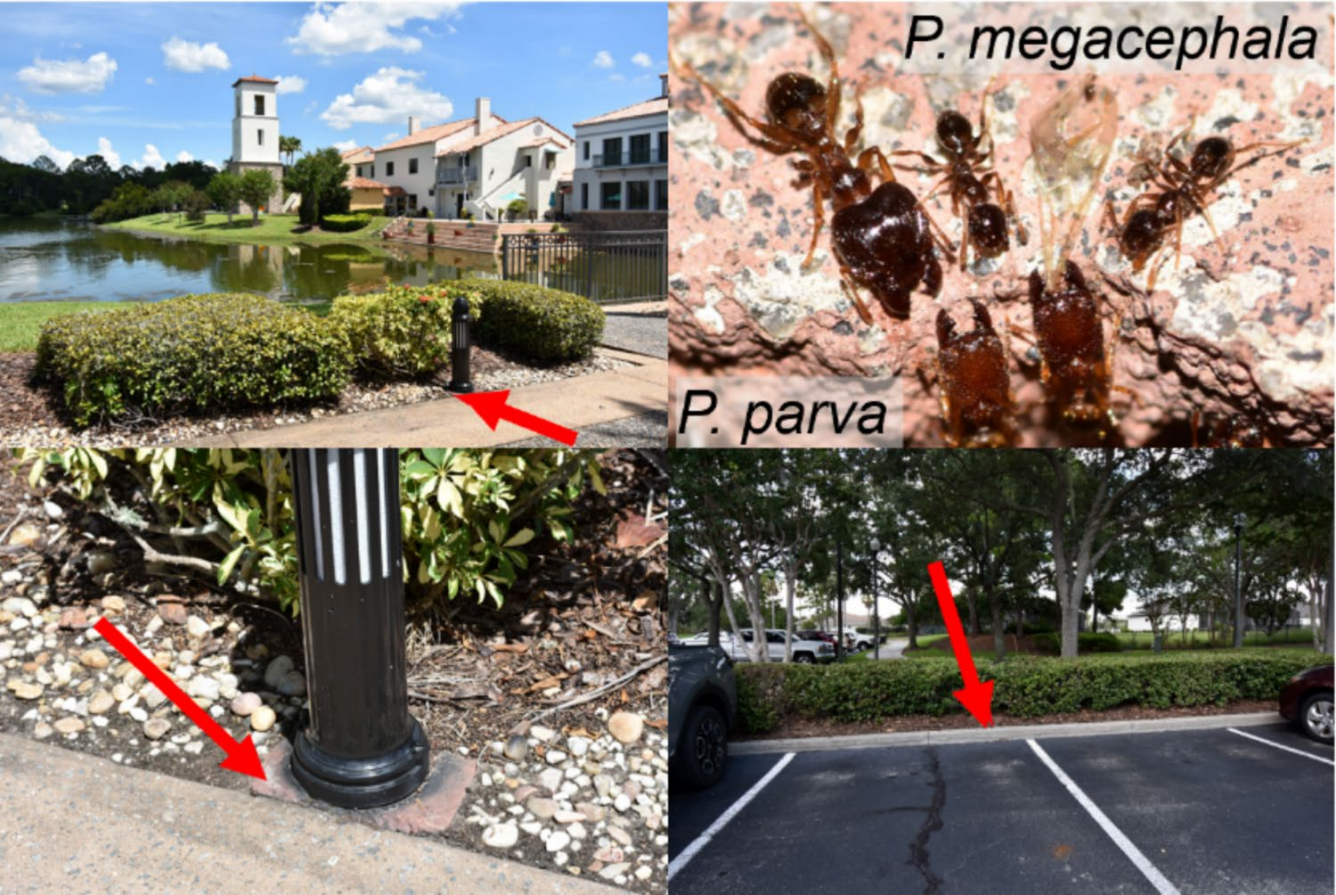
Pictures of landscape and nest area where *Pheidole parva* was collected in Central Florida. The red arrows indicate the nests that were found in cracks in asphalt parking lots and sidewalks. Pictures and observations by ASJ

Ants were collected by the authors across Florida from 2023 to 2024 using different methods including light trapping, manual collection from nests, and baiting (Table 2). Ants were preserved in 95% ethanol for genetic analysis. In addition, voucher specimens have been deposited in the Florida Collection of Arthropods, Gainesville, FL. Sampling sites included urban and suburban environments with nests found in disturbed habitats including sidewalks and cracks in asphalt (Fig. 2).

**Table 2:** Sampling information of the *Pheidole parva* specimens from Florida included in the genetic analysis

Area in Florida	Location	No. ants	Year of collection	Method trap	Collector*	Identified by*	GPS coordinates
North	Pensacola	3^G^	2023	Light	ACS	ACS	30.335, −87.364
Central	Poinciana	8^W^	2024	Manual, nests, and baits	ASJ	DBB	28.133, −81.476
South	Miami^&^	15^W^	2024	Baits	SDP	DBB	25.701, −80.333^&^

^G^ indicates gynes (alate females) and ^W^ refers to workers. *Initials for collectors and identifiers are authors of this paper. ^&^In Miami, baits were from a selection of 10 locations around the city (Porter et al. 2026) and GPS is indicated for the general survey area

### Taxonomic identification of collected specimens

Sample identification of collected specimens was conducted under a stereoscopic microscope following the taxonomic keys provided by Fisher and Fisher (2013) and Sarnat et al. (2015) (Supplementary Fig. 1). Specimens were also compared with colored ant images available on AntWeb and other relevant literature. Attention was taken to differentiate *P. parva* from morphologically similar native *Pheidole* ants. Among Florida *Pheidole*, *P. dentigula* is the species most likely to be confused with *P. parva* due to similarities in size and coloration. However, several reliable characters distinguish the two. In majors (and gynes), *P. parva* possesses pronounced reticulate rugae character on the posterior head, whereas *P. dentigula* shows only weak rugosity. Minors of *P. parva* are unique among small Florida *Pheidole* by the sheer amount of rugosity on the head compared to all small native *Pheidole* in Florida. Queens (alate females) are similarly distinguishable. Native *P. dentigula* gynes are very small, 3-mm total length, and usually fully orange to reddish, and occasionally with slightly darker gaster. In contrast, the gynes of *P. parva* are larger (4–4.5 mm) and strongly bicolored with a red head and mesosoma and a black gaster. *Pheidole parva* alates show up at developed-area lights just after dusk whereas *P. dentigula* females show up at lights before sunrise in less disturbed areas. Furthermore, *P. dentigula* is increasingly rare in urban and suburban areas across Florida.

### Global distribution data

To create the global distribution of *P. parva*, we included records from AntWeb (n.d.) (accessed 29 August 2025), literature, and our new collections that correspond to field collected specimens. We relied on published taxonomic identification by AntWeb curators, and published work (Fig. 1, Table 1). These records provide a broad view of the species’ worldwide distribution and support the confirmation of the introduction of this ant in Florida. To get a better picture of the distribution status of this ant in the state, we needed a different, region‑specific approach, for which we relied on citizen-science observations.

### Citizen-science observations

Because community‑science identifications can vary in accuracy, all iNaturalist observations used in this study were independently reviewed and verified by ACS before being included in any analyses. Occurrence records of *P. parva* were downloaded via the Global Biodiversity Information Facility (GBIF), which aggregates “Research Grade” iNaturalist records. Because the identification criteria developed for this study were tailored specifically to *Pheidole* species occurring in Florida and the southeastern United States, analyses were restricted to this region. Observations from outside this area (e.g., records from the Bahamas or a greenhouse record from Maryland) are reported for completeness but were not evaluated using our identification criteria and were not included in confidence scoring or mapping analyses.

**Fig. 3:**
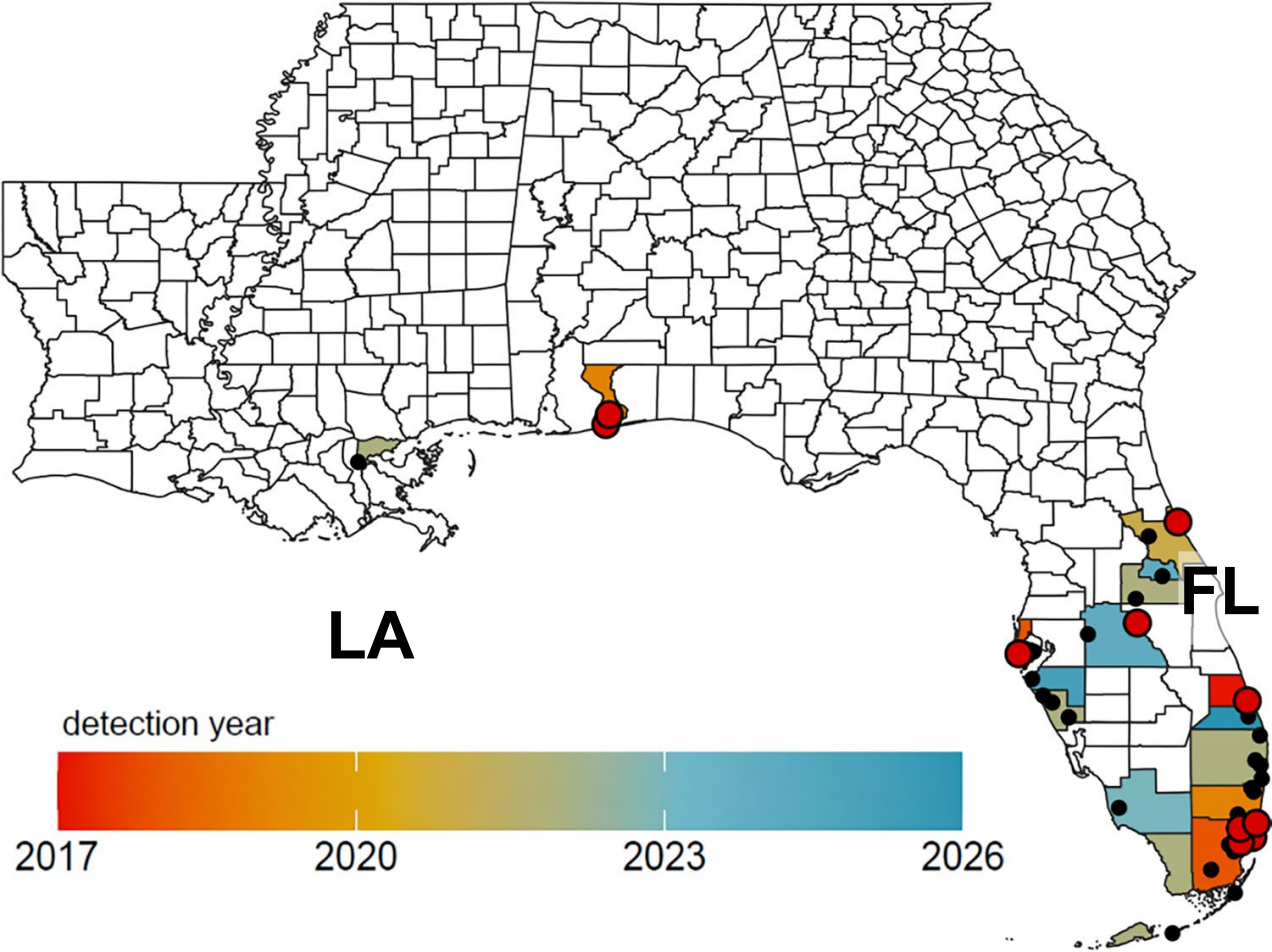
Outdoor records of *Pheidole parva* in the USA showing both specimen-confirmed occurrences as red dots from this study and iNaturalist high confidence observations as black dots. Counties are colored based on the first year of detection as shown in the figure. FL, Florida; LA, Louisiana

Each iNaturalist observation was individually reviewed and assigned to one of three data‑quality tiers (high, medium, or low confidence) based on the presence of diagnostic morphological characters visible in photographs. Observations consisting solely of male alates were excluded because males lack reliable diagnostic traits at typical iNaturalist image resolution, and no identification keys exist for male *Pheidole*. In contrast, gynes retain many of the diagnostic features used for identifying major workers allowing more reliable identification when image detail is sufficient. Most Florida observations were of dispersing female alates. Although no comprehensive key exists for the gynes of Florida *Pheidole*, identifications were guided by AntWeb specimen images, published species descriptions, known distribution patterns, habitat preferences, and alate phenology. Worker records were evaluated using the keys of Sarnat et al. (2015), supplemented by AntWeb photographs to exclude native species. Identifications were based on visible traits such as body proportions (gynes only), coloration, head shape, and the presence or absence of reticulorugose sculpture, as well as contextual information including geographic distribution, habitat association, and flight timing. Confidence tiers were assigned according to the diagnostic criteria needed to exclude other small *Pheidole* species in Florida. The set of species considered and eliminated during identification consists of the following taxa: *P. adrianoi*, *P. bicarinata*, *P. bilimeki*, *P. dentigula*, *P. flavens*, *P. floridana*, *P. metallescens*, and *P. navigans*. These species were selected based on close or superficial morphological similarity of their worker and/or gyne castes to *P. parva*. All evaluated observations and their confidence assignments are provided in Supplementary Table S1, and these records were mapped in Supplementary Fig. S2 according to their confidence‑tier color code. Only high‑confidence observations (Supplementary Table S2) are included in Fig. 3. Supplementary Fig. S3 provides visual examples of the morphological evaluation process for a gyne and workers.

### DNA extraction and PCR

All ants were preserved in 95% ethanol until processed. The 23 ants collected in Central and South Florida were workers; each worker was subjected to DNA extraction individually using the Qiagen PureGene kit according to the manufacturer’s protocol. The three ants collected in North Florida were alate females; in those cases, only the thorax of each of the females was used for DNA extraction since mating status of each alate was unknown. These DNA extractions were done following the same DNA extraction method as the worker ants. Abdomen was kept in 95% ethanol. Polymerase chain reactions (PCR) were then used to amplify the mitochondrial COI gene using forward primer LepF1 (5′-ATTCAACCAATCATAAAGATATTGG-3′) and reversed primer LepR1 (5′-TAAACTTCTGGATGTCCAAAAAATCA-3′) (Hebert et al. 2004). Each PCR reaction consisted of 25 µL total volume, which included 12.5 µL of Promega Go Taq 2X master mix (Promega Corporation, Madison, WI, USA), 1 µL of each primer, 2–4 µL of total genomic DNA, and water. The PCR’s cycling profile began with an initial denaturation at 95 °C (3 min) followed by 35 cycles of 95 °C (30 s), 48 °C (1 min), and 72 °C (2 min) and a final extension of 72 °C (10 min). PCR products were run in agarose gel to verify amplification then purified using ExoSAP-IT (USB Corporation, Cleveland, Ohio). Purified PCR products were submitted for Sanger sequencing to a service facility.

### Genetic analysis

For each ant, the forward and reverse sequences were aligned to generate a consensus sequence using Geneious Prime® 2020.2.2 (Biomatters Ltd.). The 26 new COI sequences were aligned with 18 previously published sequences of *P. parva* obtained from GenBank (Supplementary Table 3) using Geneious Prime® 2020.2.2 (Biomatters Ltd.). A final alignment of 618 bp was used to obtain a matrix of pairwise uncorrected *p*-distances (proportion of nucleotide sites at which two sequences being compared are different) among the 44 mitochondrial sequences. Their genetic relationships were estimated by constructing an unrooted neighbor-joining tree with MEGA11 (Tamura et al. 2021) (Supplementary Fig. 2). Bootstrapping was performed using 100 pseudo-replications of the dataset. The genealogical relationships among the sequences were also analyzed using the program TCS Version 1.13 (Clement et al. 2000). Sequences were deposited into GenBank with Accession numbers PX360526 to PX360551.

## Results

### Distribution records

In North America, a total of 66 observations of *P. parva* workers and 149 alates (queens and males) were compiled from iNaturalist and categorized into high-, medium-, and low‑confidence levels (Supplementary Table S1). All these observations were plotted using different colors for each confidence level on a map (Supplementary Fig. S2) showing that many of these observations overlapped geographically with the specimen‑confirmed records presented in this study, further supporting the species’ widespread distribution in the state (Fig. 3). High‑confidence iNaturalist observations were also identified in Louisiana and the Bahamas; however, these locations lack voucher specimens and therefore require further collection to confirm first detections (Supplementary Table S2).

### Temporal pattern of iNaturalist observations

**Fig. 4:**
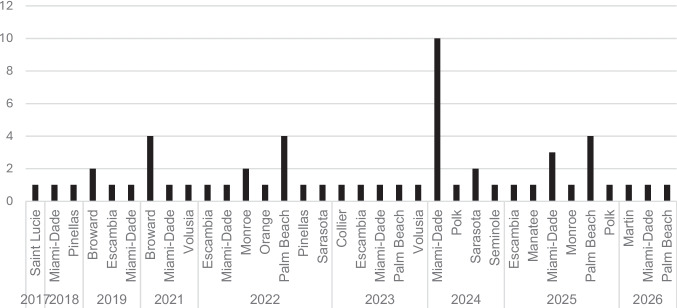
Temporal increase of curated iNaturalist observations of *Pheidole parva* in Florida from 2017 to 2026. Bars show the number of expert‑verified iNaturalist records per county for each year, only high confidence records have been plotted (see Supplementary Table S2). Although annual counts remain low (1–10 records), the number of counties reporting *P. parva* increased steadily after 2021, with detections spreading from southeastern Florida to central and northern counties. Escambia County represents an exception, with both early (2019) and recurring detections that appear tied to localized survey effort rather than part of this broader directional trend

Curated iNaturalist records from Florida assigned to the high confidence category showed a gradual but consistent increase in detections of *P. parva* between 2017 and 2026 (Fig. 4). Early records (2017–2019) were sparse and occurred primarily in a small number of southeastern counties, with the exceptions of Escambia County in the Panhandle and Pinellas County on the in west coast. Beginning in 2021, observations became more frequent and geographically widespread, with additional detections appearing in central and northern Florida. Although the number of curated observations per county per year remained low (typically 1–3 records), the increase in the number of counties reporting the species, particularly from 2021 onward, indicates a clear expansion in both reporting frequency and geographic extent.

### Genetic diversity and haplotype distribution

**Fig. 5:**
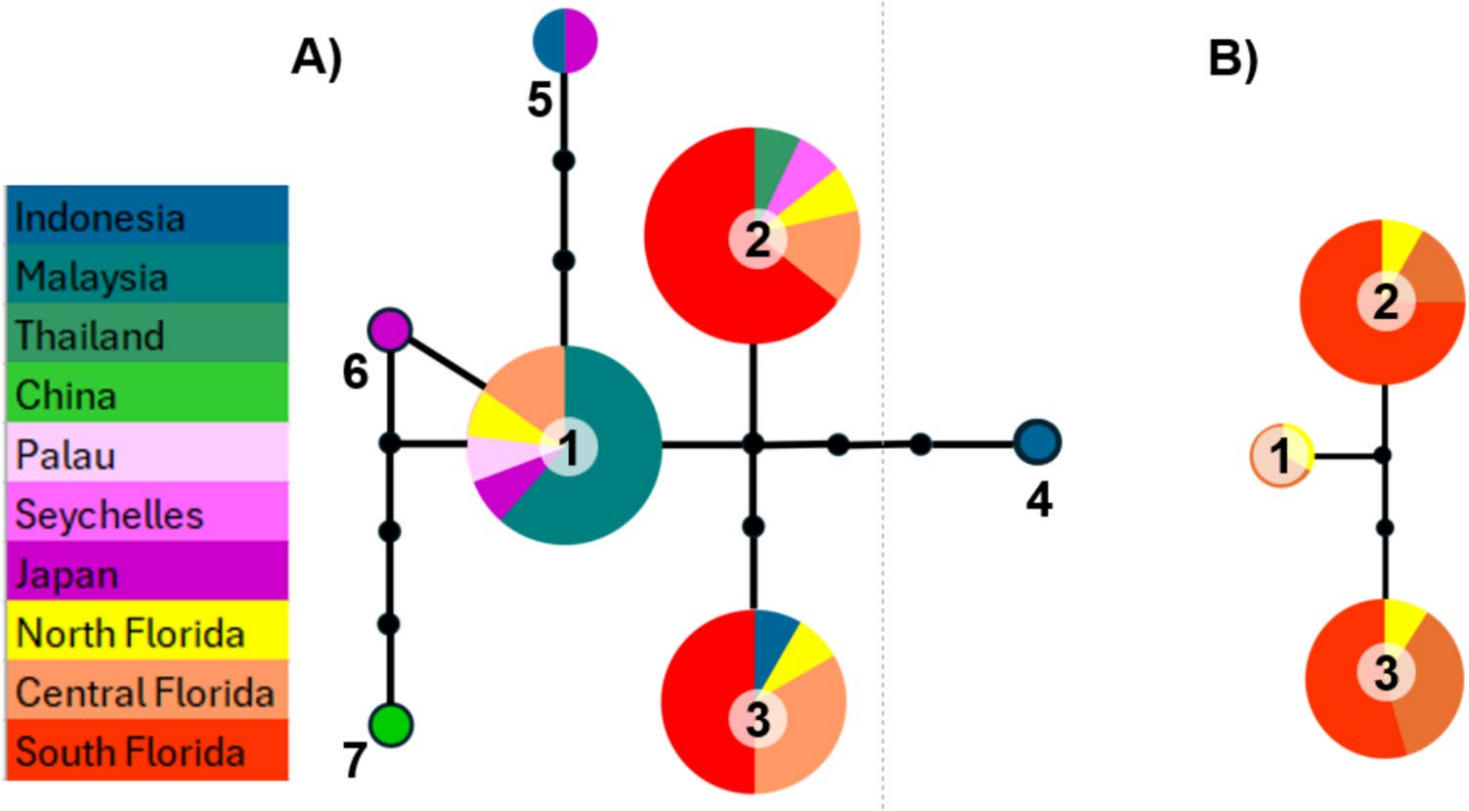
Statistical parsimony networks for the *Pheidole parva* COX1 haplotypes. **A** Network including all 44 haplotypes, with the color code legend indicating geographic origin of each sequence to the left. **B** Network including only haplotypes found in Florida. Each connecting branch represents a single mutational step, and inferred missing intermediate haplotypes are represented by filled black circles. Sizes are scaled and represent relative frequencies (see Supplementary Table S4 for absolute frequencies). GenBank details are provided in Supplementary Table S3

Analysis of the COI sequences revealed the presence of seven different haplotypes (Fig. 5). Of these, the three most common haplotypes (1, 2, and 3) were detected among specimens collected in Florida (Supplementary Table S4). In particular, haplotype 1 was detected in both North and Central Florida. Haplotypes 2 and 3 were detected across all three regions (North, Central, and South Florida). In South Florida (Miami area), haplotypes 2 and 3 were found at multiple sites scattered across Miami without any obvious pattern including locations within a few meters of each other at two sites. Although only three alate female ants were analyzed from North Florida, each exhibited a different haplotype. We note that the number of specimens available from North Florida was small, and this limited sampling should be considered when interpreting haplotype diversity in that region. All three haplotypes detected in Florida were present in samples from the native range of *P. parva*. Haplotypes 1 and 2 were additionally detected in other introduced regions, including Japan, Palau, and Seychelles. However, haplotype 3 has not yet been found in any other introduced population outside of Florida (Supplementary Table S4).

### Species identification

Morphological and genetic analyses confirmed that the specimens collected in Florida correspond to *Pheidole parva*; COI sequences obtained in this study were consistent with previously published sequences from GenBank, including specimens identified by taxonomic experts. For example, the Seychelles sequence corresponded to haplotype 2 (GenBank Accession number HQ925098) was obtained from a specimen identified Brian Fisher as *P. parva* Mayr (voucher CASENT0161023-D01) (Supplementary Table S3). Specimens from Indonesia and Japan were collected and identified by Eguchi Katsuyuki (see Supplementary Table S3 for details). In addition, morphological comparisons of major and minor workers and alate females were consistent with published description of *P. parva*.

Morphological comparisons of major and minor workers and alate females were also consistent with published descriptions of *P. parva*, and no morphological differences were observed among specimens representing haplotypes 2 and 3. Physical specimens examined in this study did not match other members of the *Pheidole rinae* complex sensu Eguchi (2007), and DNA barcoding results corroborated their identification as *P. parva*. Notably, no other species within the *P. rinae* complex is known to have established populations outside its native range. Although *P. parva* may be part of a species complex (Eguchi 2013), all specimens in this study are treated as *P. parva* following current taxonomy.

Field observations indicated that *Pheidole parva* colonies frequently occupied disturbed urban habitats, including cracks in sidewalks, parking lots, and other paved surfaces. Colonies commonly consisted of multiple nest entrances distributed across a localized area. At several sites, nests of *P. parva* were observed within short distances of nests belonging to other ant species, including *Solenopsis invicta* and *Pheidole megacephala* (Fig. 2).

## Discussion

### Recent introduction, establishment, and expansion

The presence of *P. parva* in Florida represents a recent addition to the non-native ant fauna of North America. Given the extensive historical surveys of ants in the state and the absence of earlier records supported by a detailed review of the *Pheidole* holdings at the Florida State Collection of Arthropods (Gainesville, FL), together with first observations in iNaturalist dating to 2017, it is likely that this species was introduced only recently. Despite this, its current distribution across multiple regions of Florida and its detection in neighboring regions (Louisiana and the Bahamas) suggest that this ant is becoming established in the USA and shows an expanding pattern of detections. The gradual increase in iNaturalist observations provides additional support for a recent introduction followed by expanding spread of *P. parva* in Florida (Fig. 4). Early observations were limited to a few southeastern counties, apart from early and repeated detections in Escambia County showed an early outlying detection, but from 2021 onward, records increased steadily across central and northern Florida (Fig. 4). Although curated observations per county per year remained low when only high confidence observations were included in the analysis following a more conservative approach, the increasing number of reporting counties indicates a broadening geographic footprint consistent with an expansion of its distribution. Together with the absence of historical museum specimens at the Florida State Collection of Arthropods, this temporal pattern reinforces the interpretation that *P. parva* has only recently established in Florida and is increasing its geographic distribution through the state. In Florida, recent surveys in the Miami area further highlight the rapid establishment of *P. parva* in South Florida. Porter et al. (2026) found *P. parva* to be the most frequently encountered ant species, occurring at nearly half of surveyed sites (47%), whereas *Solenopsis invicta* was detected at relatively few locations. These observations suggest that the composition of urban ant communities in South Florida is changing rapidly, although the ecological mechanisms underlying these patterns remain unclear.

The expansion of *P. parva* occurs within a broader pattern of change in Florida ant communities. Long-term analyses have documented increasing dominance of introduced ants across the state, particularly in South Florida, where non-native species now comprise a large proportion of ant occurrences (Booher et al. 2023; Porter et al. 2026). Historically, many of Florida’s most successful invasive ants have originated from the Neotropics, whereas *P. parva* represents an introduction from the Indomalayan region. Continued monitoring will be important to determine the ecological impacts of this species and whether its expansion reflects an emerging pattern of introductions from previously underrepresented biogeographic regions. In addition, the presence of three different haplotypes which are widely distributed across Florida indicates the possibility of multiple introductions from different geographic areas, or alternatively, a single source population where all three haplotypes are present. Interpretations of the genetic data should be viewed cautiously. Although our dataset includes 26 Florida specimens, it incorporates only 18 previously published sequences and relies on a single mitochondrial marker (COI). While COI is useful for confirming species identity and identifying broad patterns, it provides limited resolution for reconstructing introduction pathways or assessing population structure. Additional multilocus or genomic data, together with expanded sampling across the species’ global range, will be needed to clarify introduction history, connectivity among Florida populations, and putative source regions. Regardless of the pathway, the observed pattern and combined evidence suggest that *P. parva* has established successfully and dispersed within Florida.

### Ecology and interactions with other ants

Field observations of this species in other introduced areas suggest that this generalist forager ant can affect both natural and disturbed environments (Fischer and Fisher 2013; Sarnat et al. 2015; Carval et al. 2026). In Florida, nests were located in highly disturbed urban habitats, including cracks in pavement (Fig. 2). In these environments, *P. parva* co-occurs with other invasive ants, including *Solenopsis invicta* and *Pheidole megacephala* (San Juan et al. 2025), two of the most concerning invasive ants in Florida. During the sampling of the *P. parva* ant nests, aggressive interactions between *Pheidole megacephala* and *P. parva* were documented (Fig. 2), where *P. megacephala* approaches *P. parva* nest resulting in *P. parva* soldiers forming a line. The documented interactions with *P. megacephala* (San Juan et al. 2025) and the high frequency of occurrence reported from South Florida (Porter et al. 2026) suggest that *P. parva* can become a prominent component of urban ant communities and may be associated with shifts in local species composition in some areas. However, the mechanisms underlying these patterns, and whether they reflect competitive displacement or other indirect interactions, remain unclear and require further study. The overall ecological consequences of the spread of *P. parva* in Florida remain poorly understood. Given the high diversity of the genus *Pheidole*, particularly in the Neotropics (Wilson 2003), the establishment of a non-native *Pheidole* from the Indomalayan region underscores the importance of understanding its impact on native *Pheidole* ants, other ants, and arthropods in general, and the broader ecological impact in the Neotropics.

### Implications for detection and management

The expanding distribution of *P. parva* within Florida suggests that, in addition to natural dispersal through mating flights, human-meditated transport is likely contributing to its spread. Pathways associated with the movement of plant material such as potted ornamental plants, cut flowers, and turf are well documented for ants and represent a plausible mechanism for introduction and secondary spread. Florida’s role as a major hub for international trade, particularly through the Miami Customs District, increases the likelihood of repeated introductions of non-native species. This hub has the greatest percentage (21.8%) of insect interceptions in the USA (McCullough et al. 2006), placing Florida as a gateway to invasive insects to enter the USA. The small size of *P. parva* and its morphological similarity to native *Pheidole* species may also contribute to under detection during early stages of establishment.

The detection of *P. parva* highlights the importance of early detection and rapid response (EDRR) protocols for non-native ants in Florida. The integration of traditional taxonomic approaches with molecular tools and publicly available biodiversity data can facilitate the identification and tracking of newly introduced species. In this study, records from iNaturalist provided valuable information on the distribution and spread of this species, and these records can help in the early detection of invasive species. However, because community‑science identifications can vary in accuracy, future studies should ensure that iNaturalist observations are carefully reviewed and verified by experts before being incorporated into distributional or ecological analyses. Continued monitoring, including the use of citizen-science platforms such as iNaturalist, and genetic tools, will be essential to better understand the spread, origin, and impact of *P. parva* in North America.

## Supplementary Information

Below is the link to the electronic supplementary material.

ESM 1 (XLSX 45.4 KB)

ESM 2 (XLSX 14.2 KB)

ESM 3 (PDF 139 KB)

ESM 4 (DOCX 15.7 KB)

ESM 5 (PDF 178 KB)

ESM 6 (PDF 723 KB)

ESM 7 (PDF 338 KB)

ESM 8 (PDF 456 KB)

## Data Availability

All DNA sequence data generated in this study have been deposited in GenBank under accession numbers PX360526–PX360551. Curated iNaturalist observations and all occurrence records used in the analyses are provided in Supplementary Tables S1–S4, and links to iNaturalist and AntWeb sources are included within the manuscript.

## References

[CR1] Ahmed MZ, Weeks ENI (2025) Special issue: Invasive species records and updates. Guest editor: Muhammad Z. Ahmed. Fla Entomol 108:20250046. 10.1515/flaent-2025-0046

[CR2] AntWeb (n.d) Version 8.114. Observations of Pheidole parva. California Academy of Science, online at https://www.antweb.org. Accessed 29 Aug 2025

[CR3] Booher DB, Gotelli NJ, Nelsen MP et al (2023) Six decades of museum collections reveal disruption of native ant assemblages by introduced species. Curr Biol 33:2088-2094.e6. 10.1016/j.cub.2023.03.044 37030293 10.1016/j.cub.2023.03.044

[CR4] Carval D, Jacquelin F, Soti V et al (2026) New ant records from La Réunion Island (Hymenoptera, Formicidae). Biodivers Data J 14:e188364. 10.3897/BDJ.14.e188364 41889439 10.3897/BDJ.14.e188364 PMC13014136

[CR5] Caton BP, Dobbs TT, Brodel CF (2006) Arrivals of hitchhiking insect pests on international cargo aircraft at Miami International Airport. Biol Invasions 8:765–785. 10.1007/s10530-005-3736-x

[CR6] Clement M, Posada D, Crandall KA (2000) TCS: a computer program to estimate gene genealogies. Mol Ecol 9:1657–1659 11050560 10.1046/j.1365-294x.2000.01020.x

[CR7] Dawson W, Moser D, Van Kleunen M et al (2017) Global hotspots and correlates of alien species richness across taxonomic groups. Nat Ecol Evol 1:0186. 10.1038/s41559-017-0186

[CR8] Demetriou J, Borowiec L, Georgiadis C et al (2025) Citizen-science supplements species inventories and reveals the invasion of *Monomorium exiguum* and *Pheidole parva* (Hymenoptera: Formicidae) in Cyprus. Sociobiology 72:e11594. 10.13102/sociobiology.v72i3.11594

[CR9] Deyrup M, Davis L, Cover S (2000) Exotic ants in Florida. Trans Am Entomol Soc 1890–126:293–326

[CR10] Donisthorpe H (1946) II.— *The ants* (*Hym.* Formicidæ) *of Mauritius*. Ann Mag Nat Hist 13:25–35. 10.1080/00222934608654518

[CR11] Eguchi K, Yamane S, Zhou S-Y (2007) Taxonomic revision of the *Pheidole rinae* Emery complex. Sociobiology 50:257–284

[CR12] Fischer G, Fisher BL (2013) A revision of *Pheidole* Westwood (Hymenoptera: Formicidae) in the islands of the Southwest Indian Ocean and designation of a neotype for the invasive *Pheidole megacephala*. Zootaxa 3683:301–356. 10.11646/zootaxa.3683.4.1 25250457 10.11646/zootaxa.3683.4.1

[CR13] Forel A (1907) No. VI.-Fourmis des seychelles, amirantes, farquhar et chagos. Trans Linn Soc Lond 2nd Ser Zool 12:91–94. 10.1111/j.1096-3642.1907.tb00512.x

[CR14] Frank JH, McCoy ED (1995) Introduction to insect behavioral ecology: the good, the bad, and the beautiful: non-indigenous species in Florida. Invasive adventive insects and other organisms in Florida. Fla Entomol 78:1. 10.2307/3495661

[CR15] Hebert PDN, Penton EH, Burns JM et al (2004) Ten species in one: DNA barcoding reveals cryptic species in the neotropical skipper butterfly *Astraptes fulgerator*. Proc Natl Acad Sci U S A 101:14812–14817. 10.1073/pnas.0406166101 15465915 10.1073/pnas.0406166101 PMC522015

[CR16] Hulme PE (2009) Trade, transport and trouble: managing invasive species pathways in an era of globalization. J Appl Ecol 46:10–18. 10.1111/j.1365-2664.2008.01600.x

[CR17] King JR, Porter SD (2007) Body size, colony size, abundance, and ecological impact of exotic ants in Florida’s upland ecosystems. Evol Ecol Res 9:757–774

[CR18] Liebhold AM, Brockerhoff EG, Garrett LJ et al (2012) Live plant imports: the major pathway for forest insect and pathogen invasions of the US. Front Ecol Environ 10:135–143. 10.1890/110198

[CR19] Man L-S, Lee C-Y (2014) Structure-invading pest ants in healthcare facilities in Singapore. Sociobiology 59:241. 10.13102/sociobiology.v59i1.681

[CR20] McCullough DG, Work TT, Cavey JF et al (2006) Interceptions of nonindigenous plant pests at US ports of entry and border crossings over a 17-year period. Biol Invasions 8:611–630. 10.1007/s10530-005-1798-4

[CR21] Porter SD, Warner J, Booher DB (2026) Miami ant survey: where are the red imported fire ants? Fla Entomol 109:20250048. 10.1515/flaent-2025-0048

[CR22] San Juan A, Azémar F, Dejean A (2025) *Pheidole megacephala*: an invasive ant that raids colonies of the red imported fire ant, *Solenopsis invicta*. Ecology 106:e70113. 10.1002/ecy.70113 40357688 10.1002/ecy.70113 PMC12070350

[CR23] Sarnat EM, Fischer G, Guénard B, Economo EP (2015) Introduced *Pheidole* of the world: taxonomy, biology and distribution. ZooKeys 543:1–109. 10.3897/zookeys.543.6050 10.3897/zookeys.543.6050 PMC4714327 26798286

[CR24] Schifani E, Massaad M (2026) Seven species in seven days: new additions to the ant fauna Hymenoptera, Formicidae) of Lebanon. J Insect Biodivers Syst 12. 10.48311/jibs.12.02.255

[CR25] Sharaf MR, Fisher BL, Al Dhafer HM et al (2018) Additions to the ant fauna (Hymenoptera: Formicidae) of Oman: an updated list, new records and a description of two new species. Asian Myrmecol 10:e010004. 10.20362/am.010004

[CR26] Sharaf MR, Wetterer JK, Mohamed AMA et al (2024) Filling gaps in global myrmecology: ants of the Kingdom of Bahrain (Hymenoptera: Formicidae). J Nat Hist 58:1705–1786. 10.1080/00222933.2024.2388791

[CR27] Skelley PE, Buss EA, Allen JS et al (2025) Adventive arthropods in Florida as reported by the Florida Department of Agriculture and Consumer Services, Division of Plant Industry, 1990–2023. Fla Entomol 108:20240027. 10.1515/flaent-2024-0027

[CR28] Stein BA, Kutner LS, Adams JS et al (eds) (2000) Precious heritage: the status of biodiversity in the United States. Oxford University Press, Oxford, New York

[CR29] Tamura K, Stecher G, Kumar S (2021) MEGA11: molecular evolutionary genetics analysis version 11. Mol Biol Evol 38:3022–3027. 10.1093/molbev/msab120 33892491 10.1093/molbev/msab120 PMC8233496

[CR30] Terayma M, Tomioka Y, Kimura G, Tanikawa T (2021) An exotic ant, *Pheidole parva* (s.l.), found in a port area in Honshu, Japan. Urban Pest Manag 11:75–79. 10.34348/urbanpest.11.2_75

[CR31] Volk M, Hoctor T, Nettles B et al (2017) Florida land use and land cover change in the past 100 years. Florida’s climate: changes, variations, & impacts. Florida Climate Institute

[CR32] Wilson EO (2003) *Pheidole* in the New World: a dominant, hyperdiverse ant genus. Harvard University Press

[CR33] Wong MK, Economo EP, Guénard B (2023) The global spread and invasion capacities of alien ants. Curr Biol 33:1–6. 10.1016/j.cub.2022.12.020 36610395 10.1016/j.cub.2022.12.020

