# Supplementary material for: First Records and Expanding Distribution of a Small Big-Headed Ant, Pheidole parva, in Florida, USA
Source: Neotrop Entomol. 2026 Jul 21;55(1):66. doi: 10.1007/s13744-026-01416-4 (PMC13388651; doi:10.1007/s13744-026-01416-4)
Supplement: Supplementary file 3 — (PDF 139 KB) [file 13744_2026_1416_MOESM3_ESM.pdf]

Supplementary Table S3. Information of all the sequences included in the analysis per geographic region, sampling location, year of collection, their GenBank Accession number, the taxonomy reported by the authors of the sequences, and the authors listed in the GenBank Accession.

Geographic region	Sampling Location	Year	GenBank Accession #	Taxonomy from reference	Reference in GenBank Accession
Native Range					
Indomalaya region	Indonesia; Lombok Island	1998	AB772024	*Pheidole cf. sauteri*	Eguchi, K. and Oguri, E., Japan, direct submission
	Indonesia; Central Java	2002	AB772025	*Pheidole cf. sauteri*	Eguchi, K. and Oguri, E., Japan, direct submission
	Indonesia; Batam Island	2011	AB772034	*Pheidole cf. sauteri*	Eguchi, K. and Oguri, E., Japan, direct submission
	Malaysia; Penang	2003	AB772026	*Pheidole cf. sauteri*	Eguchi, K. and Oguri, E., Japan, direct submission
	Malaysia; Penang	2023	OR073712	*Pheidole parva*	Chan, F. S., Malaysia, direct submission
	Malaysia; Penang	2023	OR073713	*Pheidole parva*	Chan, F. S., Malaysia, direct submission
	Malaysia; Penang	2023	OR073714	*Pheidole parva*	Chan, F. S., Malaysia, direct submission
	Malaysia; Penang	2023	OR073715	*Pheidole parva*	Chan, F. S., Malaysia, direct submission
	Malaysia; Penang	2023	OR073717	*Pheidole parva*	Chan, F. S., Malaysia, direct submission
	Malaysia; Penang	2023	OR073718	*Pheidole parva*	Chan, F. S., Malaysia, direct submission
	Malaysia; Penang	2023	OR073720	*Pheidole parva*	Chan, F. S., Malaysia, direct submission
	Thailand; Chiang Mai	2012	AB772019	*Pheidole cf. sauteri*	Eguchi, K. and Oguri, E., Japan, direct submission
	China; Guangdong; Guangzhou	2024*	PP440201	*Pheidole parva*	Huang, Y., China, direct submission
Introduced Range					
East Asia	Japan; Ryukyus; Okinawa Island	2012	AB772023	*Pheidole cf. sauteri*	Eguchi, K. and Oguri, E., Japan, direct submission
	Japan; Ogasawara Island	2012	AB772028	*Pheidole cf. sauteri*	Eguchi, K. and Oguri, E., Japan, direct submission
	Japan; Ogasawara Island	2012	AB772029	*Pheidole cf. sauteri*	Eguchi, K. and Oguri, E., Japan, direct submission
Micronesia	Palou Island	2008	KJ141795	*Pheidole sp.*	(Economo et al., 2015)
SWIO	Seychelles	2010	HQ925098	*Pheidole parva*[&]	International Barcode of Life (iBOL), direct submission
North America	US, Florida, Pensacola	2023	PX360534	*Pheidole parva*	This report
	US, Florida, Pensacola	2023	PX360535	*Pheidole parva*	This report
	US, Florida, Pensacola	2023	PX360536	*Pheidole parva*	This report
	US, Florida, Poinciana	2024	PX360526	*Pheidole parva*	This report
	US, Florida, Poinciana	2024	PX360527	*Pheidole parva*	This report
	US, Florida, Poinciana	2024	PX360528	*Pheidole parva*	This report
	US, Florida, Poinciana	2024	PX360529	*Pheidole parva*	This report
	US, Florida, Poinciana	2024	PX360530	*Pheidole parva*	This report
	US, Florida, Poinciana	2024	PX360531	*Pheidole parva*	This report
	US, Florida, Poinciana	2024	PX360532	*Pheidole parva*	This report
	US, Florida, Poinciana	2024	PX360533	*Pheidole parva*	This report
	US, Florida, Miami	2024	PX360538	*Pheidole parva*	This report
	US, Florida, Miami	2024	PX360539	*Pheidole parva*	This report

Supplementary Table 3. Continued.

Geographic region	Sampling Location	Year	GenBank Accession #	Taxonomy from reference	Reference in GenBank Accession
Introduced Range					
	US, Florida, Miami	2024	PX360540	*Pheidole parva*	This report
	US, Florida, Miami	2024	PX360541	*Pheidole parva*	This report
	US, Florida, Miami	2024	PX360542	*Pheidole parva*	This report
	US, Florida, Miami	2024	PX360543	*Pheidole parva*	This report
	US, Florida, Miami	2024	PX360544	*Pheidole parva*	This report
	US, Florida, Miami	2024	PX360545	*Pheidole parva*	This report
	US, Florida, Miami	2024	PX360546	*Pheidole parva*	This report
	US, Florida, Miami	2024	PX360547	*Pheidole parva*	This report
	US, Florida, Miami	2024	PX360548	*Pheidole parva*	This report
	US, Florida, Miami	2024	PX360549	*Pheidole parva*	This report
	US, Florida, Miami	2024	PX360550	*Pheidole parva*	This report
	US, Florida, Miami	2024	PX360551	*Pheidole parva*	This report

SWIO: Southwest Indian Ocean
*Date submitted to GenBank
[&]voucher CASENT0161023-D01

Economo, E.P., Klimov, P., Sarnat, E.M., Guénard, B., Weiser, M.D., Lecroq, B., Knowles, L.L., 2015. Global phylogenetic structure of the hyperdiverse ant genus *Pheidole* reveals the repeated evolution of macroecological patterns. Proc. R. Soc. B Biol. Sci. 282, 20141416. https://doi.org/10.1098/rspb.2014.1416
