# Supplementary material for: First Records and Expanding Distribution of a Small Big-Headed Ant, Pheidole parva, in Florida, USA
Source: Neotrop Entomol. 2026 Jul 21;55(1):66. doi: 10.1007/s13744-026-01416-4 (PMC13388651; doi:10.1007/s13744-026-01416-4)
Supplement: Supplementary file 4 — (DOCX 15.7 KB) [file 13744_2026_1416_MOESM4_ESM.docx]

**Supplementary Table S4.** Global geographic distribution of mitochondrial DNA haplotypes (1 to 7) found in *Pheidole parva* based on the COX1 gene.

| **Geographic region** | ***1*** | ***2*** | ***3*** | ***4*** | ***5*** | ***6*** | ***7*** | **Totals** |
| --- | --- | --- | --- | --- | --- | --- | --- | --- |
| Indonesia |  |  | 1 | 1 | 1 |  |  | 3 |
| Malaysia | 8 |  |  |  |  |  |  | 8 |
| Thailand |  | 1 |  |  |  |  |  | 1 |
| China |  |  |  |  |  |  | 1 | 1 |
| Palau | 1 |  |  |  |  |  |  | 1 |
| Seychelles |  | 1 |  |  |  |  |  | 1 |
| Japan | 1 |  |  |  | 1 | 1 |  | 3 |
| North Florida | 1 | 1 | 1 |  |  |  |  | 3 |
| Central Florida | 2 | 2 | 4 |  |  |  |  | 8 |
| South Florida |  | 9 | 6 |  |  |  |  | 15 |
| Totals | 13 | 14 | 12 | 1 | 2 | 1 | 1 | 44 |
