# Supplementary material for: First Records and Expanding Distribution of a Small Big-Headed Ant, Pheidole parva, in Florida, USA
Source: Neotrop Entomol. 2026 Jul 21;55(1):66. doi: 10.1007/s13744-026-01416-4 (PMC13388651; doi:10.1007/s13744-026-01416-4)
Supplement: Supplementary file 5 — (PDF 178 KB) [file 13744_2026_1416_MOESM5_ESM.pdf]

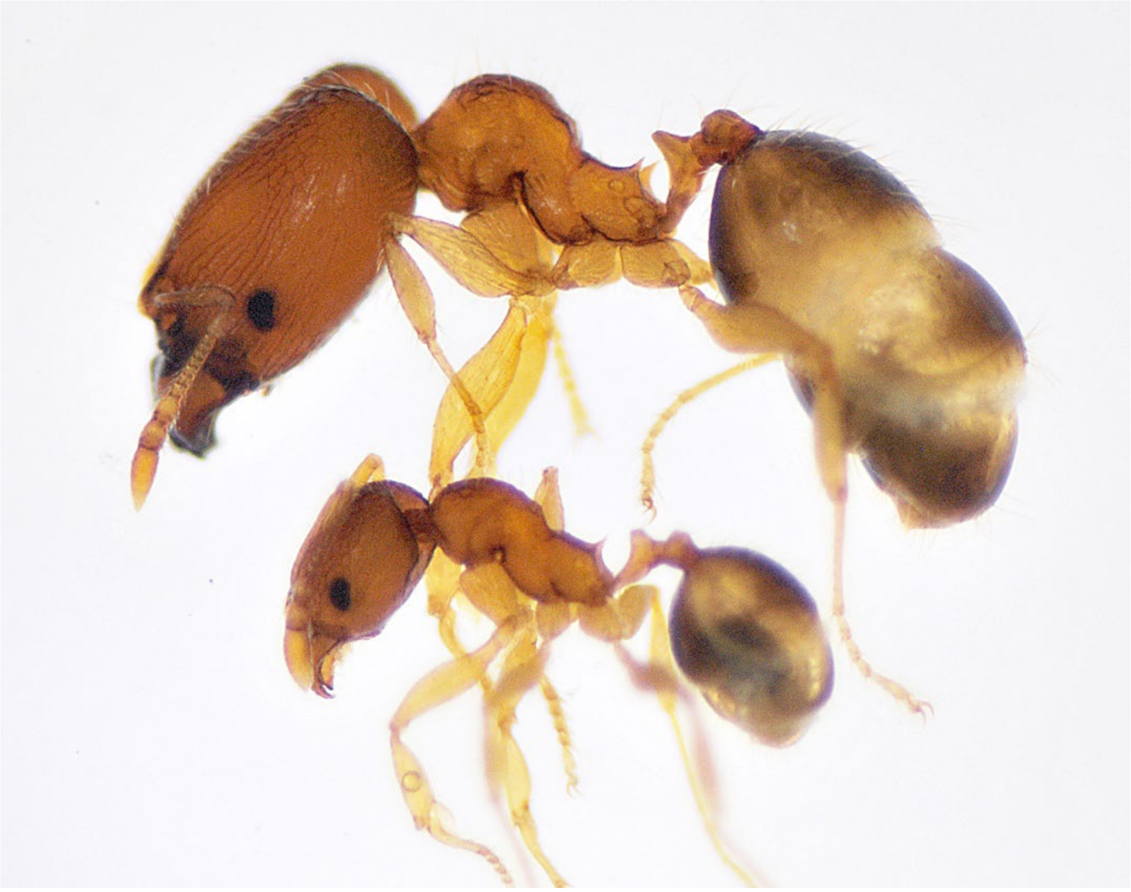

Supplementary Fig. S1. Major and minor *Pheidole parva* workers collected at Miami bait site #39 in South Florida.
