# Supplementary material for: First Records and Expanding Distribution of a Small Big-Headed Ant, Pheidole parva, in Florida, USA
Source: Neotrop Entomol. 2026 Jul 21;55(1):66. doi: 10.1007/s13744-026-01416-4 (PMC13388651; doi:10.1007/s13744-026-01416-4)
Supplement: Supplementary file 6 — (PDF 723 KB) [file 13744_2026_1416_MOESM6_ESM.pdf]

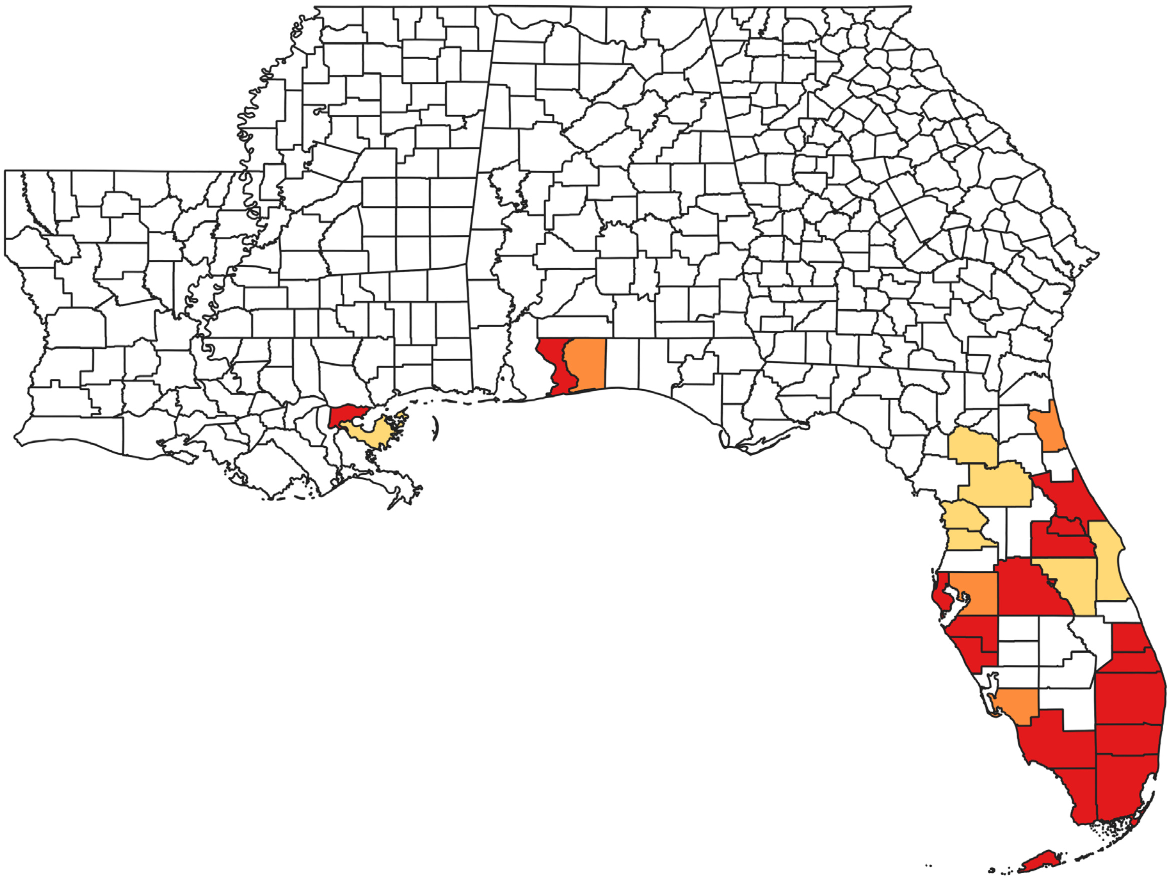

Supplementary Fig. S2. Spatial distribution of all expert-evaluated iNaturalist observations of *Pheidole parva* in Florida from Supplementary Table S1. Observations are color-coded by identification confidence tier: red = high confidence, orange = medium confidence, and yellow = low confidence. Confidence levels were assigned based on visible diagnostic morphological traits (see Materials and Methods and Supplementary Table S1).
