# Supplementary material for: First Records and Expanding Distribution of a Small Big-Headed Ant, Pheidole parva, in Florida, USA
Source: Neotrop Entomol. 2026 Jul 21;55(1):66. doi: 10.1007/s13744-026-01416-4 (PMC13388651; doi:10.1007/s13744-026-01416-4)
Supplement: Supplementary file 7 — (PDF 338 KB) [file 13744_2026_1416_MOESM7_ESM.pdf]

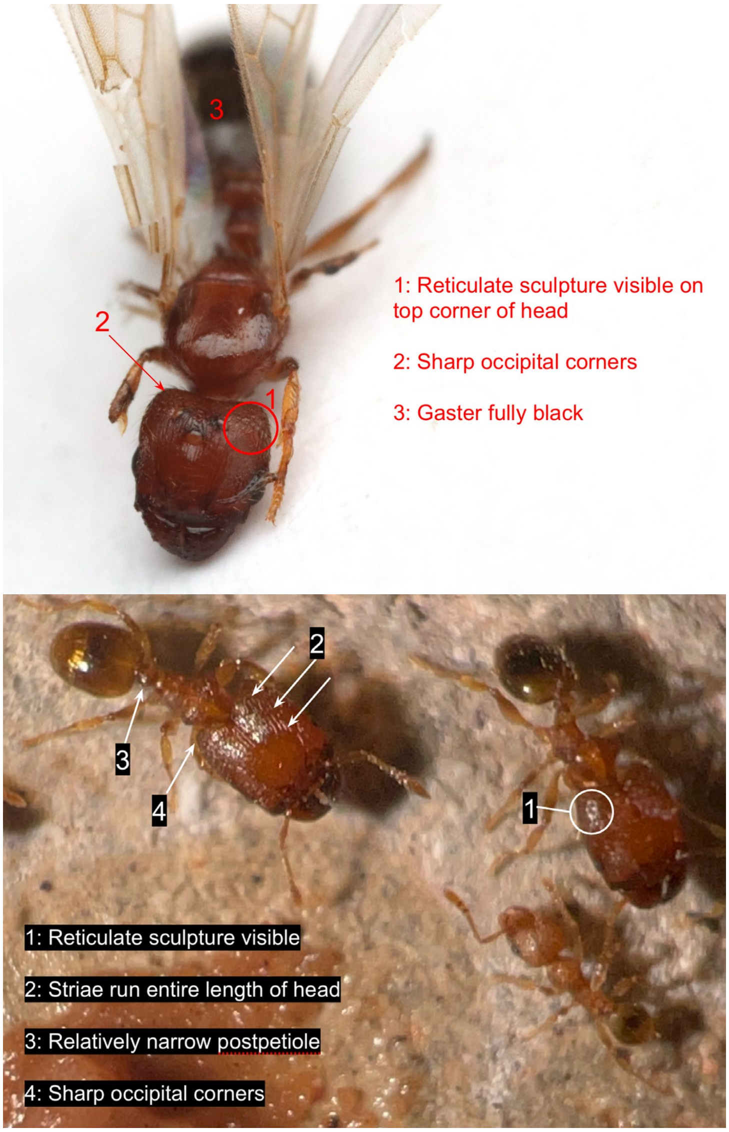

Supplementary Fig. S3. Representative iNaturalist observations for *Pheidole parva* demonstrating how diagnostic morphological features were evaluated using the expert-verification approach developed for this study and guided by published taxonomic keys (see Materials and Methods). These observations were assigned to the high-confidence category and used in the distribution maps and analyses. (A) Gyne (iNaturalist Observation 284823233; gbifID 5292021953), showing a strongly bicolored body, characteristic head shape, and reticulate sculpture on the posterior head. (B) Workers (iNaturalist Observation 265700564; gbifID 5291791583), illustrating diagnostic head rugosity and other traits used to distinguish *Pheidole parva* from similar Florida *Pheidole* species.
