# Supplementary material for: First Records and Expanding Distribution of a Small Big-Headed Ant, Pheidole parva, in Florida, USA
Source: Neotrop Entomol. 2026 Jul 21;55(1):66. doi: 10.1007/s13744-026-01416-4 (PMC13388651; doi:10.1007/s13744-026-01416-4)
Supplement: Supplementary file 8 — (PDF 456 KB) [file 13744_2026_1416_MOESM8_ESM.pdf]

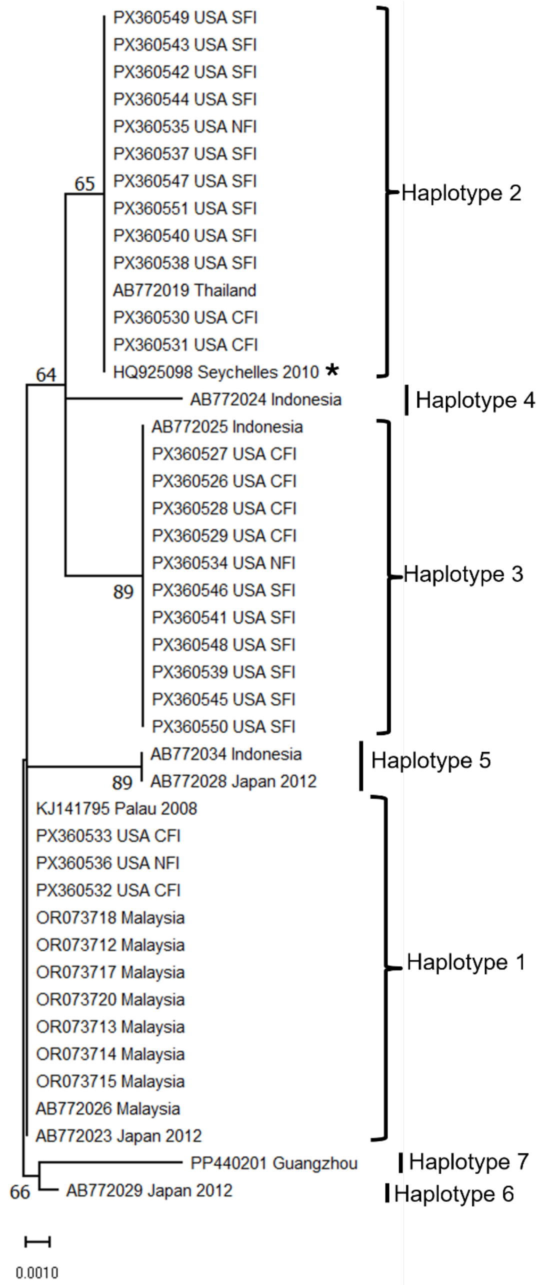

Supplementary Fig. S4. Neighbor-Joining (NJ) tree constructed based on the *P. parva* *p*-distances found among the ants analyzed in this study. Numbers on branches represent bootstrap support values (100 replicates). All ambiguous positions were removed for each sequence pair (pairwise deletion option). There was a total of 618 positions in the final dataset. This is an unrooted tree. Sequences obtained from ants collected in Florida are labelled: USA NFl, for North Florida, CFl, Central Florida, and SFl, South Florida. Haplotypes are indicated 1 to 7 as described in Figure 3 and Table 3. *Sequence from Seychelles specimens correspond to voucher CASENT0161023-D01, collected by B. L. Fisher et al. and identified as *Pheidole parva* Mayr.
